# Diagnostic Challenges in Retinitis Pigmentosa: Genotypic Multiplicity and Phenotypic Variability

**DOI:** 10.2174/138920211795860116

**Published:** 2011-06

**Authors:** Susie Chang, Leah Vaccarella, Sunday Olatunji, Colleen Cebulla, John Christoforidis

**Affiliations:** 1 Retina Division, Havener Eye Institute, The Ohio State University College of Medicine, Columbus, Ohio, USA

**Keywords:** Retinitis pigmentosa, heterogeneity, clinical manifestation, phenotypic variation, genotype-phenotype correlation, genetic testing.

## Abstract

Retinitis pigmentosa (RP) is a heterogeneous group of inherited retinal disorders. Diagnosis can be challenging as more than 40 genes are known to cause non-syndromic RP and phenotypic expression can differ significantly resulting in variations in disease severity, age of onset, rate of progression, and clinical findings. We describe the clinical manifestations of RP, the more commonly known causative gene mutations, and the genotypic-phenotypic correlation of RP.

## INTRODUCTION

Retinitis pigmentosa (RP) represents a heterogeneous group of inherited retinal dystrophies characterized by abnormalities of photoreceptors and retinal pigment epithelium that lead to progressive retinal degeneration, atrophy and ultimate visual loss. RP affects 1 in 3500 to 5000 people worldwide [1, 2]. Classification of retinitis pigmentosa reveals two main groups: “typical” or “non-syndromic” RP in which disease manifestations are confined to the eye and “complicated” or “syndromic” RP in which patients have associated non-ocular findings. The latter group includes Usher syndrome, Bardet-Biedl syndrome, Refsum syndrome, Neuronal ceroid lipofuscinosis or Batten disease, Alstrom disease, Bassen-Kornzweig syndrome, Kearns-Sayre syndrome, and Mucopolysaccharidoses [1]. The differential diagnosis of retinitis pigmentosa is vast, and the combination of multiple causative genes and broad range of clinical severity has made both diagnosis and prognosis challenging. Herein, we discuss the genotypic multiplicity and phenotypic variability found in typical or non-syndromic retinitis pigmentosa.

## CLINICAL MANIFESTATIONS AND PHENOTYPIC VARIABILITY

Retinitis pigmentosa is characterized by nyctalopia, constricted visual fields, bone spicule pigmentation of the fundus, and photoreceptor cell dysfunction. Clinical presentation and severity varies with inheritance pattern. Individuals with X-linked RP generally present early whereas those with autosomal dominant RP have a later onset. The same genetic mutation can also produce variable expression patterns not only between families, but also within families. Incomplete penetrance and variable expressivity may be due to interactions between genes or their environment as there are many factors that can interfere with gene expression from transcription to protein activation.

### Ocular Manifestations

Funduscopic findings include arteriolar narrowing, fine intraretinal pigmentation, and loss of retinal pigment epithelium (RPE) typically in the mid- and far-periphery. With progression of primarily rod photoreceptor degeneration, there is increasing loss of RPE pigment with intraretinal clumping of melanin appearing as coarse clumps in the classic bone spicule configuration [1]. With time, retinal vascular attenuation and waxy pallor of the optic nerve become more apparent. Occasionally, deep white dots at the level of the RPE, secondary to nonspecific pigment epithelial degeneration, arise, a feature of retinitis punctata albescens [1]. In typical RP, the central retina is relatively spared until very advanced stages with the onset of foveomacular atrophy.

In the vitreous, fine colorless particles comprised of free melanin pigment granules, pigment epithelium, uveal melanocytes, and macrophage-like cells may occur usually early in the course of typical RP before funduscopic changes are clinically apparent [1]. Optic nerve head drusen are also common without any diagnostic or prognostic significance. Later manifestations of RP include cataract, usually a central posterior sub-capsular type with a clear nucleus. Symptomatically, it tends to obstruct the central visual field and exacerbate photophobia. Central visual acuity can also be affected due to macular edema, the result of intracellular swelling of Müller cells and breakdown of the blood-retinal barrier. Patients with advanced RP can also develop pseudo-Coats’ disease, a peripheral exudative vasculopathy associated with telangiectatic vessels, serous retinal detachment, and retinal lipid deposition.

Both eyes are comparably affected along a phenotypic spectrum ranging from retinitis pigmentosa sine pigmento with normal fundus findings and photoreceptor degeneration, to sector RP affecting 1-2 quadrants (usually infero-nasal) of the fundus, to diffuse retinal pigmentary changes.

### Visual Field

Progressive loss of peripheral visual field is characteristic of RP. Visual field testing is a subjective examination mapping one’s central and peripheral vision. Kinetic perimetry, such as Goldmann visual field testing, utilizes a moving test target of variable size against a uniformly illuminated background and tests out to 90^o^ temporally. In early RP, there is a patchy mid-peripheral field loss 20-25^o^ from fixation. With disease progression, a mid-peripheral ring scotoma develops and enlarges more rapidly toward the periphery than centrally [1].

### Dark Adaptation

Dark adaptation testing is useful in the evaluation of nyctalopia, which occurs early in RP. Recovery of photoreceptor sensitivity after light exposure is dependent on the regeneration of bleached photo-pigments. After adapting to darkness, the sensitivity to light increases; that is, the threshold response to light decreases. Patients adapt to 30-60 minutes of darkness and then detect the dimmest light possible. In normal dark adaptation the transition between the faster cone function and slower rod function, or cone-rod break, occurs after 5 minutes. Abnormalities associated with RP are variable and include a delayed cone-rod break, an elevated final threshold, and a prolonged recovery to final threshold. In advanced RP, the final rod threshold can be 3 or more log units greater than normal [3, 4]. In individuals with rhodopsin mutations, the late rod portion of dark adaptation is affected, whereas early rod and cone adaptation are normal [4].

### Visual Acuity

Visually acuity is usually maintained until very late in the course of RP unless macular atrophy or cystoid macular edema is present. Visual acuity is variable, but in general vision loss is characterized as less severe in individuals with autosomal dominant RP than with autosomal recessive RP. Patients with X-linked RP typically have the worst visual prognosis [1, 5].

### Color Vision

Macular cone function can be assessed with color vision testing. Deficiency in blue cone function (acquired tritanopia) is characteristic of advanced RP. Individuals with vision worse than 20/30 and areas of macular atrophy correspond to poorer performance on color vision tests such as the Nagel anomaloscope and Farnsworth-Munsell 100-hue test. Those with autosomal dominant RP correlate to better color vision testing than those with other inheritance types [5].

### Electroretinography

Conventional or full-field electroretinography (ERG) objectively demonstrates the overall functional status of the photoreceptors by measuring retinal electrical potential after light stimulation. A dim blue light (dark adapted) single flash induces a rod response and a flickering (30 Hz) white light elicits a cone response. Early and severe impairment of pure rod responses occurs in RP with a dramatic decrease in amplitude and implicit times of both a-waves (photoreceptor) and b-waves (signaling by second-order bipolar cells). Advanced RP patients have extinguished rod and cone responses [1, 6].

Whereas full-field ERG will reveal abnormalities when more than 20% of the retina is affected, multifocal ERG can localize affected areas of retinal dysfunction. In RP, multifocal ERG demonstrates a significant reduction in the paracentral retina compared to the central retina [7]. There is no correlation between multifocal ERG pattern and inheritance type [8].

### Electro-Oculography

Electro-oculography (EOG) reflects a global outer retina and RPE function. When ERG findings are abnormal, EOG is also abnormal. In RP patients, its main utility may be in the evaluation of carriers with questionable funduscopic and ERG findings. In X-linked RP, EOG measurements are extinguished in RP patients and subnormal in RP carriers. EOG abnormalities have been found more often in RP patients over the age of 40 years [9].

### Optical Coherence Tomography

Optical Coherence Tomography (OCT) is a non-invasive testing modality providing morphological information of the retina. It assesses the vitreoretinal interface, retinal contour and thickness, presence of intraretinal or subretinal fluid, and photoreceptor layer and RPE status. Compared to time-domain OCT, Fourier-domain OCT provides greater axial resolution and scanning speed allowing for more detail of the structural changes within the retinal layers, specifically the outer segment, inner segment, outer nuclear layer, as well as the RPE. The relationship between retinal structure and visual function has been studied in RP patients utilizing Fourier-domain OCT and automated perimetry as well as microperimetry [10, 11]. In RP patients, photoreceptor thickness as demonstrated on OCT decreases with loss of visual field sensitivity [10].

OCT is also useful as a screening tool in the detection of sub-clinical cystoid macular edema (CME) [12, 13]. Hajali *et al*. reported a 38% prevalence of CME in at least 1 eye. Although not statistically significant, the prevalence of CME was found to be 52% for autosomal dominant RP, 39% for autosomal recessive RP, 39% for isolated RP, and none for X-linked RP [13].

## GENOTYPIC HETEROGENEITY IN RETINITIS PIGMENTOSA

The molecular genetics of retinitis pigmentosa are complex. RP is associated with an expanding number of newly identified gene mutations, which can be sporadic or familial with multiple modes of transmission. Genes associated with RP code for proteins that play important roles in phototransduction, the visual transduction cascade, and photoreceptor transcription and structure [1]. In addition, while many different genes may result in a similar disease phenotype, there is wide variation in phenotypic results within families sharing the same genetic mutation. RP has been associated with a variety of inheritance patterns and some genes may have more than 1 mode of transmission. Roughly 20-25% of cases are autosomal recessive, 15-20% of cases are autosomal dominant, 10-15% of cases are X-linked, and the residual majority of cases making up 40-55% appear to be sporadic or are unable to be classified [2]. To date, 17 autosomal dominant, 24 autosomal recessive, and 6 X-linked RP loci have been mapped [14]. Table **1** summarizes the known genes implicated in RP [1, 2, 14-40].

## AUTOSOMAL DOMINANT RP

Autosomal dominant retinitis pigmentosa represents approximately 15-20% of RP cases [2]. It is generally the least severe form of RP, with later onset and tendency to retain good visual acuity. The three most common gene mutations involved in autosomal dominant RP include *RHO* (rhodopsin), *RP1* (oxygen-regulated proteins), and *RDS* (RDS/Peripherin). 

### *RHO* Mutations

Rhodopsin is a transmembrane photo-pigment located in the discs of rod photoreceptor outer segments. Rhodopsin is responsible for light absorption and initiation of the visual phototransduction cascade. Over 100 mutations in the rhodopsin gene (*RHO*) have been identified and linked to RP with over 30% of cases involving autosomal dominant RP [2, 41, 42]. Rhodopsin, a key transmembrane structural component, makes up approximately 85% of the protein content of rod photoreceptor outer segments [43]. It has three domains, the intradiscal, the transmembrane, and the cytoplasmic domain. Mutations have been identified in each of the three domains. Sandberg *et al*. linked RP phenotypic severity with the location of the rhodopsin mutation involved [44]. Patients with mutations in the intradiscal domain tend to have better visual prognosis than those with transmembrane domain mutations, while patients with cytoplasmic domain mutations have the worst visual function [44].

The most common mutation in the rhodopsin gene found in the United States in the Pro23His mutation. This mutation is located on the rhodopsin intradiscal domain and accounts for 10-15% of autosomal dominant RP in American Caucasians [45]. The phenotype associated with the Pro23His mutation is relatively mild when comparing patient visual fields and ERG amplitudes [44, 46].

Mutations found in the rhodopsin cytoplasmic domain carries a worse visual prognosis and more severe phenotype and may be due to the important cellular transport functions of this domain [2, 46]. In particular, several disease mutations have been found in the 347 codon of the rhodopsin cytoplasmic domain. These mutations, including the relatively common Pro347Leu mutation, have been associated with a comparatively poor visual prognosis [47].

### *RP1* Mutations

The *RP1* gene, located at 8q12.1, has been associated with approximately 5-10% of autosomal dominant retinitis pigmentosa cases [1]. Expression of RP1 appears to be regulated by retinal oxygen levels and has been shown to encode a photoreceptor protein involved in the correct stacking of photoreceptor outer segments [48, 49]. The gene contains 4 exons. Four *RP1 *mutations account for the majority of *RP1* associated retinitis pigmentosa. These are R677X, Q679X, 2433del5bp, and 2435del4bp, with R677X and Q679X responsible for up to 60% of all *RP1* mutations. All four of the major *RP1* mutations result in truncation of the RP1 protein and cause photoreceptor degeneration. However, between families and individuals with the same mutation great variation in phenotypic manifestations exists, and environmental as well as genetic factors may be involved [2].

### *RDS* Mutations

The *RDS* gene located on chromosome 6p21.1-cen encodes for the protein peripherin, or RDS/Peripherin, and is responsible for an estimated 5-10% of autosomal dominant retinitis pigmentosa [1]. Studies have shown that RDS/Peripherin accumulates in the discs of photoreceptor outer segments and is important for photoreceptor structure and stability [50]. The RDS/Peripherin protein is made up of four transmembrane domains and three loops, with the most severe phenotypic variants arising from mutations within the intradiscal loop between the third and fourth transmembrane domains [51]. Mutations of RDS/Peripherin protein are numerous and associated with a wide range of phenotypes including other autosomal dominant macular dystrophies [52-56].

## AUTOSOMAL RECESSIVE RP MUTATIONS

Autosomal recessive RP is the most common inherited form of the disease comprising 20-25% of cases. Autosomal recessive RP is often associated with earlier age of symptom onset and more severe phenotype than autosomal dominant RP. Most of the genes associated with autosomal recessive RP are very rare. Several of the more common gene mutations found in autosomal recessive RP include *ABCA4*, *RPE65*, *PDE6A* and *PDE6B*, and *USH2A*, many of which are also associated with other autosomal recessive retinal disorders [1].

### *ABCA4* Mutations

The photoreceptor cell-specific ATP-binding cassette transporter (*ABCA4*) gene is located on chromosome 1p21-p13 and has been implicated in up to 5% of autosomal recessive retinitis pigmentosa cases [57]. *ABCA4* codes for an important transmembrane transport protein found in the discs of photoreceptor outer segments [58]. It is made up of 6 transmembrane helices, an exocytoplasmic domain within the photoreceptor discs and a cytoplasmic domain. The normal ABCA4 transporter is thought to bind retinylidene-phosphatidylethanolamine (N-retinylidene-PE) in the discs and transport it to the cytoplasmic side. Here N-retinylidene-PE breaks down into all-trans-retinal, a form of vitamin A that can be used in the visual cycle [59]. *ABCA4* mutations have been associated with autosomal recessive RP as well as Stargardt macular dystrophy, fundus flavimaculatus, cone rod dystrophy, and even some cases of age-related macular degeneration [60].

### *RPE65* Mutations

The *RPE65* gene has been associated with autosomal recessive retinitis pigmentosa as well as autosomal recessive Leber congenital amaurosis (LCA). *RPE65* accounts for about 2% of all autosomal recessive RP and is responsible for 6-16% of cases of LCA. *RPE65* is associated with metabolism of retinoids involved in the visual cycle [10].

## X-LINKED MUTATIONS

X-linked retinitis pigmentosa accounts for approximately 10 to 15% of all retinitis pigmentosa and tends to be the most severe form of the disease with the earliest onset and the most rapid progression. Affected males develop visual symptoms early in life, often manifesting symptoms of night blindness before age 20, progressing to significant visual impairment by the 4^th^ decade. Affected females usually have a less severe phenotype, likely due to random X-inactivation [2]. Two genes have been implicated in the majority of cases of X-linked retinitis pigmentosa, *RPGR* and *RP2*.

### *RPGR* Mutations

The retinitis pigmentosa GTPase regulator (*RPGR*) gene is located on chromosome Xp21.1 and accounts for approximately 70-90% of cases of X-linked RP [1]. *RPGR* is composed of 19 exons and codes for a protein made up of 815 amino acids. To date, 77 gene mutations have been identified and are mainly localized in exons 1-14 and ORF15. Most of these mutations result in early termination of translation [2]. The function of *RPGR* is currently unknown, but gene products have thus far been associated with protein transport and intracellular trafficking. Mutations of *RPGR* have been associated with a variety of diseases including X-linked recessive and X-linked dominant RP, as well as X-linked cone-rod dystrophy and X-linked macular degeneration [24]. Specific X-linked RP *RPGR* mutations are often found only in single families, making genotypic-phenotypic correlations difficult [2].

### *RP2* Mutations

*RP2* is located on chromosome Xp11.3 and accounts for roughly 10-20% of cases of X-linked RP [1]. The *RP2* gene consists of 5 exons encoding a 350 amino acid protein. Most of the *RP2* mutations commonly found in X-linked RP result in sequence truncation or missense mutations. According to a recent study by Jayasundera *et al*., *RP2* mutations in X-linked RP have been associated with patients of younger age, poorer visual acuity, high myopia, and fundus appearance similar to choroideremia without corresponding choroideremia gene mutations [61].

## GENOTYPE-PHENOTYPE CORRELATIONS

### Age of Onset

Early onset RP is diagnosed around 2 years of age. The causative genes include *RPE65*, *CRB1*, and *TULP1* [5, 62-64]. Late onset RP, diagnosed when symptoms of RP develop in mid-life, is typically autosomal dominant in inheritance [2, 65]. The *CRX* gene is involved in late-onset autosomal dominant RP [66].

### Fundus Appearance

In general absence or scarcity of pigment deposits is more frequent in individuals with myopia due to retinal pigment atrophy or RP sine pigmento and does not necessarily reflect the severity of disease [5]. Mutations in several genes are associated with specific fundus findings. Mutations in the *RHO* and *PRPF31* genes are involved in sector RP [5, 67]. The *CRB1* gene is associated with para-arteriolar retinal pigment epithelium preservation [68]. Retinitis punctata albescens is seen in *RLBP1* gene mutations [69]. Severe macular atrophy in RP involves *RDS* and *CRX* genes [5, 52]. A mutation in the *CRB1* gene likely in association with other genetic or environmental factors has been found in individuals with Coats’-like exudative vasculopathy [70].

## GENETIC TESTING IN RP

Genetic testing for RP could potentially provide more specific disease characterization and prognostic information. More importantly, gene-specific treatment is becoming more promising as seen with ongoing clinical trials for Leber Congenital Amaurosis [2, 71-73]. Molecular testing, however, remains to be defined in RP due to its genetic heterogeneity with no single mutation accounting for more than 10% of RP patients [2].

Current conventional screening technology includes conformation sensitive gel electrophoresis, denaturing high performance liquid chromatography, and direct DNA sequencing. In cases of unknown inheritance pattern, current screening methods are costly, time-consuming, and labor-intensive as it involves 500 exons consisting of over 120,000 base pairs. Moreover, the rate of false positives is relatively high due to polymorphic variants. Thus, screening for patients with an unknown inheritance pattern is not a feasible or practical option. The task of screening individuals with a known inheritance pattern may be somewhat less arduous but remains inefficient. Screening for X-linked recessive RP involves 2 genes comprising of 24 exons with 7000 base pairs. Twenty genes comprised of 337 exons with 75,000 base pairs are screened in autosomal recessive RP, the most common inheritance pattern, whereas 15 genes divided into 135 exons and including 38,000 base pairs are screened in autosomal dominant RP [68]. Unfortunately, conventional screening is not 100% sensitive as some mutations go undetected. Furthermore, non-pathogenic DNA variations that lead to silent mutations or mutations that do not significantly alter the protein may be detected.

DNA micro-array technology, a high-throughput screening tool, uses a disease chip containing all known disease associated alleles [74-76]. It is faster, less costly, and theoretically can be updated to screen for new genes or mutations. Advantages of this technology compared to conventional techniques include the miniaturized size of the arrays and the ability to assess thousands of genes at one time with minimal tissue sample [77]. Furthermore, the large number of probes on a single micro-array slide can include replicates and controls to improve sensitivity, specificity, and accuracy.

Micro-array as a diagnostic screening tool is limited in that only known mutations are detected, thus overlooking rare and novel mutations. A significant number of RP cases are caused by deep intronic mutations, which would not be detected as well. Micro-array screening in combination with sequence analysis could potentially detect novel mutations [76, 78, 79]. With continued development, micro-array technology in RP could allow for accurate prognostic and therapeutic insights.

Currently, 42 genes can be tested by centers throughout the world for autosomal dominant, autosomal recessive, and X-linked RP. GeneTests provides a directory of these laboratories [http://www.ncbi.nlm.nih.gov/sites/GeneTests/?db=GeneTests].

## CONCLUSION

The correlation between genotype and phenotype is not completely evident in RP. Causative genes continue to be identified making diagnosis difficult. Moreover, the exact cause of penetrance and expressivity are still not well elucidated. Developing an adequate screening test especially without a known inheritance pattern will continue to remain a challenge. The importance cannot be understated as identification of these genes could potentially lead to novel treatments or gene therapy trials.

## Figures and Tables

**Table 1:** Genes in Retinitis Pigmentosa

Inheritance	Gene Symbol	Locus	Gene Name / Protein Name	Function
AD	CRX	19q13.32	Cone-rod otx-like homeobox TF	Photoreceptor cell transcription factor
AD	FSCN2	17q25	Retinal fascin homolog 2	Structure of photoreceptor cells
AD	IMPDH1	7q32.1	Inosine monophosphate dehydrogenase 1	Regulation of cell growth
AD	PRPF3/HPRP3	1q21.2		Pre-mRNA splicing factor
AD	PRPF31/RP11	19q13.42		Pre-mRNA splicing factor
AD	PRPF8	17p13.3		Pre-mRNA splicing factor
AD	RDH12	14q24.1	Retinol dehydrogenase 12	Visual transduction cascade
AD	ROM1	11q12.3	Rod outer-segment membrane protein1	Photoreceptor structure
AD	RP7/PRPH2	6p21.1	Peripherin 2	*
AD	RP7/RDS	6p21.2	Peripherin/RDS	Photoreceptor structure
AD	RP9	7p14.3	RP9 protein	*
AD	RP17/CA4	17q22	Carbonic anhydrase IV	Pre-mRNA splicing factor
AD	RP31/TOPORS	9p21.1	Topoisomerase I binding protein	*
AD	RP33/SNRNP200	2q11.2	Small nuclear ribonucleoprotein	*
AD	RP35/SEMA4A	1q22	Semaphorin 4A	Neuroretinal development
AD	RP37/NR2E3	15q23	Nuclear receptor subfamily 2, E3	Transcription factor
AD	RP42/KLHL7	7p15.3	Kelch-like 7 protein	Protein degradation
AD	RP48/GUCA1B	6p21.1	Guanylate cyclase activating protein	*
AD/AR	NRL	14q11.2	Neural retina lucine zipper	Photoreceptor cell transcription factor
AD/AR	PDC	1q25-32.1		Visual transduction cascade
AD/AR	RHO/RP4/RP5	3q22.1	Rhodopsin	Phototransduction cascade
AD/AR	RP1	8q12.1		Photoreceptor transcription factor
AD/AR	RP50/BEST1	11q12.3	Bestrophin	RPE transmembrane chloride channel
AR	ABCA4	1p22.1	ATP binding cassette transporter	Catabolic functions in the retina
AR	CERKL/RP26	2q31-q33	Ceramide kinase	Ceramide metabolism
AR	CNGA1/RP49	4p12	Rod cGMP gated channel protein	Visual transduction cascade
AR	CNGB1/RP45	16q13	Rod cGMP gated channel protein	Visual transduction cascade
AR	CRB1/RP12	1q31.3	Crumbs protein homolog 1	Transcription factor
AR	IMPG2	3q12.3	Interphotoreceptor matrix proteoglycan 2	*
AR	LRAT	4q32.1	Lecithin retinol acyltransferase	Retinoid metabolism
AR	MERTK/RP38	2q13	Mer receptor tyrosine kinase	Disc shedding
AR	PDE6A/RP43	5q33.1	Rod cGMP phosphodiesterase	Visual transduction cascade
AR	PDE6B/RP40	4p16.3	Rod cGMP phosphodiesterase	Visual transduction cascade
AR	PDE6G	17q25.3	Rod cGMP phosphodiesterase	Visual transduction cascade
AR	RBP3	10q11.22	Retinol binding protein 3	Retinoid transport
AR	RGR/RP44	10q23.1	RPE-retinal G protein coupled receptor	Retinoid metabolism
AR	RLBP1	15q26.1	Retinaldehyde-binding protein 1	Retinoid metabolism
AR	RP22	16p12.1p12.1		*
AR	RP25	6cen-q15		*
AR	RP28	2p16-p11	Family with sequence similarity 161, A	*
AR	RP29	4q32-q34		*
AR	RP32	1p21.2-p13.3		*
AR	RP36/PRCD	17q25.1	Progressive rod-cone degeneration protein	*
AR	RP41/PROM1	4p15.32	Prominin 1	*
AR	RP46/IDH3B	20p13	NAD(+) specific isocitrate dehydrogenase 3 B	*
AR	RP51/TTC8	14q32.11	Tetratricopeptide repeat domain 8	*
AR	RP54/C2ORF71	2p23.2	Chromosome 2 open reading frame 71	*
AR	RPE65/RP20	1q31.2	RPE65	Retinoid metabolism
AR	SAG/RP47	2q37.1	Rod arrestin	Visual transduction cascade
AR	SPATA7	14q31.3	Spermatogenesis associated protein 7	*
AR	TULP1/RP14	6p21.31	Tubby-like protein 1	Vesicular transport
AR	USH2A/RP39	1q41	Usherin	Retinal development, cell adhesion
AR	ZNF513	2p23.3	Zinc finger protein 513	*
XL	RP2	Xp11.23	Homology with human cofactor C	Protein folding
XL	RP6	Xp21.3-21.2		*
XL	RP23	Xp22		*
XL	RP24	Xq26-27		*
XL	RP34	Xq28-qter		*
XL	RPGR/RP15	Xp21.1	Retinitis pigmentosa GTPase regulator	Protein transport

* Function not fully identified.
